# Parenting through the eyes of mothers with substance use disorder: Implications for treatment and related services

**DOI:** 10.1016/j.drugalcdep.2025.112782

**Published:** 2025-07-08

**Authors:** Brittany T. Smith, Danielle D. Davidov, Meghan Gannon, Caroline P. Groth, Alfgeir L. Kristjansson

**Affiliations:** a Department of Pediatrics, University of Pittsburgh School of Medicine, 4401 Penn Avenue, Pittsburgh, PA 15224, United States; b Department of Social and Behavioral Sciences, West Virginia University School of Public Health, 64 Medical Center Drive, Morgantown, WV 26505, United States; c College of Nursing, Thomas Jefferson University Sidney Kimmel Medical College, 1233 Locust Street, Philadelphia, PA 19107, United States; d Department of Epidemiology and Biostatistics, West Virginia University School of Public Health, 64 Medical Center Drive, Morgantown, WV 26505, United States

**Keywords:** Qualitative, Maternal substance use, Parenting

## Abstract

**Background:**

Maternal substance use can negatively impact parenting. However, most research on this topic come from epidemiological studies. Thus, the purpose of this study is to qualitatively explore how mothers receiving substance misuse treatment feel their substance use disorder (SUD) has influenced their experiences of motherhood and parenting while also exploring their perception of how their children may have been impacted by their SUD

**Methods:**

35 one-on-one semi-structured interviews with mothers receiving substance use treatment were conducted and analyzed using thematic analysis.

**Results:**

Four themes emerged from the analysis: 1) Battle between recovery and motherhood, 2) Challenges being fully present as a parent, 3) Parenting within government and legal systems, and 4) Impacts on children. Mothers mostly reported that their SUD did negatively impact their children in numerous ways. However, their SUD was also reported to be a barrier to parenting that brought unique challenges.

**Conclusion:**

Parenting and SUD are interrelated and can influence one another. To best serve mothers with SUD and their children this interconnectedness must be considered.

## 1. Introduction

Globally, there has been an increase in substance use among women of childbearing age (Wouldes and Lester, 2023). The number of women in the United States (US) with an opioid use disorder (OUD) at the time of childbirth more than quadrupled with rates going from 1.5 per 1000 delivery hospitalizations in 1999–6.5 in 2014 (Haight, 2018). In West Virginia (WV) the increase was more than 50 times higher, with rates going from.6 per 1000 delivery hospitalizations in 2000–32.1 in 2014. Between 2015 and 2019, 28.4 % of women who reported past year substance use had at least one child residing with them (Jones et al., 2024). Problematic household substance use, which can include maternal substance use disorder, is an adverse childhood experience (ACE), which is a potentially traumatic experience that occurs before the age of 18 (Felitti et al., 1998). Other defined ACEs include various experiences of abuse, neglect, and household dysfunction (Felitti et al., 1998). ACEs have been linked to adverse health consequences later in life, such as increased risks for substance use disorder (SUD), heart attacks, depression, and early mortality (Dube et al., 2003; Felitti et al., 1998; Rod et al., 2020). Children exposed to maternal substance use disorder therefore may be at elevated risk for experiencing ACEs and other forms of maltreatment (Austin et al., 2020; Kepple, 2017; Moreland et al., 2021; Smith et al., 2021; Walsh et al., 2003). One study found that children of mothers in SUD treatment experienced 3.9 ACEs on average, which was significantly more than children who did not have mothers with SUD (Smith et al., 2021). To date, research has not explored mothers understanding of their children’s ACEs or how their substance use may have contributed to children’s experiences with ACEs. However, gaining understanding of mother’s perceptions of their children’s ACE could be an important contribution towards the tailoring of parental interventions. The data from mother narratives may also provide insight into other adversities that are not categorized as ACEs by the traditional measurement that may impact their children’s lives.

Maternal SUD likely impacts children’s outcomes because of how the symptoms of the disorder influence parenting (Milligan et al., 2020). Research has found that substance use can negatively impact various aspects of parenting (Guyon-Harris et al., 2023; Hatzis et al., 2017). For example, mothers with SUD were less likely to have positive parenting attributes such as, positive parent-child relationships and high levels of understanding, confiding, and consistency when compared to mothers without substance use impairment (Arria et al., 2012). Mothers with Opioid Use Disorder (OUD), a specific form of SUD, have been found to have less parental involvement and lower levels of maternal sensitivity, structuring, and non-intrusiveness when compared to mothers without OUD (Romanowicz et al., 2019; Salo et al., 2010; Suchman and Luthar, 2000). Some studies linked methamphetamine use in parents to a variety of negative emotional responses that range from high irritability to apathy (Brown and Hohman, 2006; Dyba et al., 2019; Dyba et al., 2019). However, substance use alone does not inevitably equate to poor parenting behaviors. There are variations in the literature on substance use and parenting, with some studies reporting that generally parents with SUD provide appropriate care for their children and that there are other attributes that play a role in the relationship between poor parenting behaviors and substance use (Conners-Burrow et al., 2013; Grant et al., 2011; Hans et al., 2022). Those factors, that often coexist in the lives of women with SUD, include childhood trauma, mental illness, high exposure to intimate partner violence, and lower socioeconomic status (Chassin et al., 2019; Lloyd, 2018; Magnuson and Duncan, 2019; Savage et al., 2019). The potential harms from these often-coexisting factors are difficult to disentangle from the parenting challenges that stem from substance use. Therefore, it is important not to solely focus on the potential impact that substance use may have on parenting. For example, poverty increases parenting stress which negatively impacts family function and parent-child relationships (Ho et al., 2022). Additionally, parental substance use, exposure to domestic violence, and parental mental health challenges are interrelated experiences that are often exacerbated by poverty and collectively and negatively affect parenting behaviors (Adjei et al., 2022; Barrett et al., 2024). Research has found that negative children’s outcomes are augmented when these factors occur together (Barrett et al., 2024; Kedzior et al., 2024).

Systematic barriers may also play a role in the parenting behaviors of mothers with SUD. The criminal justice and child protection systems can pose significant challenges to families impacted by maternal SUD. In numerous studies mothers with SUD have cited these systems as barriers to receiving adequate SUD treatment (Barnett et al., 2021; Brogly et al., 2018; Choi et al., 2022). Studies have also found that women that felt unfairly treated by the CPS system also often felt it hindered their potential for recovery (Choi et al., 2022). The risks of prosecution by the criminal justice system and fear of losing custody over their children are additional barriers to mothers access to SUD treatment (Choi et al., 2022). Successful substance use treatment can lead to positive changes in parenting skills (Moreland and McRae-Clark, 2018). When mothers with SUD are fearful of accessing services provided by CPS and the criminal justice systems, it could negatively impact their potential for recovery and therefore the potential for positive outcomes in their children.

While epidemiological studies have linked mothers’ substance use to poor parenting and negative children’s outcomes, underlining the need for appropriate interventions, the mother’s perspective on this relationship is largely missing, but crucial, for suitable tailoring of interventions and services. Intervention development requires the perspective of those who they are intended for to ensure that it will address the most pressing needs and be appropriately engaging (Yardley et al., 2015). Furthermore, mothers with SUD are a population that often face judgment and stigma that they ignore their children’s needs (Mayes, 2023). Such assumptions highlight a need for qualitative research that contributes to improved understanding of the lived experiences of mothers with SUD. Providing in-depth accounts of these experiences can lead to the reduction of stigma and in the long run empower this population by bringing their voices to the forefront of the literature. Such reporting may counteract common power imbalances such as experiences by mothers with SUD with healthcare providers and law enforcement agents while also calling attention to the needs and challenges of this population (Stutterheim and Ratcliffe, 2021). To date, qualitative studies have provided evidence that combats the stigma faced by mothers with SUD. For example, findings suggest that motherhood is a motivator to seek substance use treatment, while stigma and judgment are reasons for avoiding treatment and other services that could benefit both mothers and their children (Adams et al., 2021; Bakos-Block et al., 2022; Barnett et al., 2021). Qualitative studies have primarily examined how being a mother impacts treatment engagement, with study samples predominantly consisting of mothers engaging in parenting programs (Barnett et al., 2021; Kerwin et al., 2014; Moreland et al., 2020). Additionally, studies have begun to reveal that mothers often feel guilty about how their substance use has impacted their children (Adams et al., 2021; Herriott et al., 2024). However, very few studies have focused on the inter-influence of SUD and parenting, specifically how mothers explain how their SUD may have impacted their parenting. Therefore, the purpose of this study is to explore how mothers receiving substance use treatment, primarily for Opioid Use Disorder and Methamphetamine Use Disorder, feel their SUD has influenced their experiences of motherhood and parenting while also exploring their perception of how their children may have been impacted by their SUD. We aimed to gain a deeper understanding of how maternal substance use has affected motherhood and parenting to inform the treatment of parenting women with SUD and provide services that will help them.

## 2. Methods

### 2.1. Approach

This study employed a fundamental qualitative description approach to answer the research questions (Sandelowski, 2000). This qualitative approach is preferred when a straightforward summary of participants’ experiences using their own words is needed, rather than a highly interpretive recollection (Neergaard et al., 2009). Qualitative description is useful when describing perceptions that are poorly understood or when little is known about the phenomenon (Doyle et al., 2019). Since this study population often faces stigma, judgement, and inaccurate representation of their experiences, we chose an approach that would most accurately present the phenomenon in a way that prioritizes the voices of mothers with SUD with minimal interpretation.

### 2.2. Recruitment

For this analysis we recruited mothers who were part of a larger mixed-methods study that sought to examine adverse childhood experiences in mothers in SUD treatment and their children while describing their experiences as mothers with SUD. The inclusion criteria for the overall study were 1) being between the ages of 18–64, 2) receiving SUD treatment from a participating substance use treatment center in West Virginia, and 3) being assigned female sex at birth. The age of the child at the time of data collection, stage of treatment, and number of prior treatment engagements did not impact study eligibility. Participants who did not have biological children were excluded from the study. A target sample for the qualitative study of 30 participants was established based on prior literature to ensure thematic saturation was reached (Hennink and Kaiser, 2022). However, after reaching our intended goal of 30 participants, five additional participants from the larger study asked to be interviewed about their experiences and were included in the qualitative sample as well. This study protocol was approved by the West Virginia University Institutional Review Board (Protocol Number: 2212690650).

### 2.3. Data collection

Mode of data collection was via short quantitative survey and semi-structured individual interviews. Interviews were conducted between August 2023 and December 2023 either in-person at the respective treatment facility or via Zoom. Participants were recruited from four outpatient centers and three residential treatment centers in West Virginia. All participants completed the one-time anonymous Qualtrics survey on an iPad. Upon completion of the survey participants were asked if they would be interested in a one-time semi-structured interview. Those who were interested and available then were taken to a private room at the facility for the interview. Those who were interested but were not available at the time of survey completion were instructed to contact the PI to schedule a virtual interview via Zoom. Before conducting the interview, the PI obtained informed consent verbally to minimize identity disclosure risk. The sensitive nature of the questions was also discussed in detail with each participant, and it was emphasized that they could refuse to answer any questions or stop the interview at any time.

### 2.4. Survey

All study participants completed the survey, which inquired about sociodemographic characteristics, mothers’ ACE exposure, parenting stress, and their relationship with their children. The survey also included questions about their children’s ACE exposure and emotional and behavioral challeneges. No identifiable information was collected during the survey. However, participants were invited to provide their contact information to the PI if they wanted to be contacted about future research opportunities related to the study. Those who were interested were contacted via a telephone call when it was time to conduct member checking. A total of three participants were contacted and agreed to be a part of the member checking process.

### 2.5. Interview

The average interview took 45–60 min to complete. An interview guide was used to frame the conversation (Appendix A). The participants were asked questions about their childhood experiences, their experiences with becoming a mother and parenting, and their children’s lives and how it has potentially been impacted by substance use. Upon completing the interviews, and in line with the study IRB protocol, participants were provided with a list of resources to use if they found any questions distressing. Each participant received a $25 gift card for completing the interview. All interviews were audio recorded and transcribed verbatim, with identifying information removed before analysis.

### 2.6. Data analysis

Descriptive statistics were obtained for sociodemographic variables, mother’s ACE score and children’s ACE score. R-Studio was used for all statistical analyses. The qualitative data was analyzed using inductive thematic analysis in MAXQDA software (Clarke and Braun, 2017). The coding team consisted of the PI, a doctoral candidate with experience in qualitative research (BS), a doctoral level research assistant (HL), two doctoral students (JC, JJ), with supervision provided by experienced qualitative researchers (DD, MG). The PI developed a preliminary code with definitions for each code to share with the other coders. While the PI analyzed all interview transcripts, two coders coded 11 transcripts, while one coded 12 transcripts. Peer debriefing and member-checking were utilized to increase the trustworthiness and credibility of the results. The coders took time to review all the transcripts they were assigned and used the code book to analyze two transcripts. The PI then met with each analyst after they pilot-tested the preliminary code book to make any necessary changes or updates. When each coder completed their analysis, they met with the PI to discuss any discrepancies or differences in coding. The PI developed emerging themes based on all coders’ analyses and presented them to the coding team, where the themes were finalized. The themes were presented to 3 participants who were interested in being more involved in the research study via a Zoom video call to ensure they accurately represented their experiences. During this session, the participants were presented with descriptions of each theme and quotes corresponding to the theme. The study results comply with the Consolidated Criteria for Reporting Qualitative Research Guidelines.

### 2.7. Positionality and reflexivity

In qualitative research, the lived experiences of the participants are central to understanding the phenomenon of interest. However, the researcher’s experiences and beliefs can influence the results, as “the researcher is the research instrument” (Dodgson, 2019). For this reason, the primary researcher must acknowledge their experiences and beliefs. For this study, the PI had her own personal experiences with SUDs and ACEs. Both of her parents had SUD or used substances throughout her childhood, with her mother periodically entering substance use treatment. The PI was also able to experience her mother’s journey with long-term recovery. Having parents who engaged in substance use greatly impacted the life of the PI. She recalls many negative experiences that were difficult for her to cope with, but she also remembers the deep love and longing she had for her family. She also remembers the positive role models in her life, including her mother, who demonstrated determination and persistence, and her father, who was consistently the primary caregiver. This shaped her motives and goals throughout her life as she longed to support not only mothers receiving SUD treatment but also their children. From her personal experiences, she felt that there were very few services that supported children experiencing parental substance use. Therefore, she decided to explore if this was true and what services were most needed. She began to question if the lack of services and interventions available to children of substance-using parents resulted in her being diagnosed with Major Depressive Disorder and Generalized Anxiety Disorder.

Being the child of parents with SUD meant that there were many situations and environments she found herself in throughout her life that could be unsafe, even in her own home. This is what led to her experiencing ACEs. While the PI remembers the ACE she experienced, she did not know if these experiences were either positive or negative, for her, they were just a way of life. However, in her adult life, she recognized the harm these events had caused her. She struggled greatly during her adolescence and young adulthood with interpersonal relationships, particularly with family, and varying mental health symptoms. These struggles caused significant disruptions in her life that she was able to overcome with proper services and interventions. Having such close personal encounters with the subject of this research study naturally influences the research questions, how the PI perceived the circumstances of the participants, and the interpretation of the results. Therefore, various strategies were employed to control for this potential bias. For example, three additional coders were used to help code the transcripts and develop the themes. Qualitative experts were also consulted about appropriately sharing the PI’s lived experiences with the participants. Also, the PI did an extensive literature review to better understand the experiences of others instead of entirely relying on her own experiences.

## 3. Results

Thirty-five participants completed the interview sessions. Descriptive statistics for all study variables can be found in Table 1. The mean age for the sample was 35.9 years, with the majority reporting White race (91.4 %). A little over half of the participants reported a household income of less than $25,000 a year. Approximately two-thirds of the sample reported more than a high school education. The mean ACE score for the participants was 5.4, while the mean ACE score reported by the interviewees for their children was 3.42. Parental separation/divorce was the most reported ACE (85.7 %), followed by household substance use (77.1 %) (see Table 2).

The following themes emerged during qualitative thematic analyses: 1) Battle between recovery and motherhood, 2) Challenges being fully present as a parent, 3) Parenting within government and legal systems, which contained child protection services and separation as a subtheme, and 4) Impacts on children, which contained the subtheme of Age-dependent consequences. Additional quotes to support the presented themes are presented in Table 3.

### 3.1. Battle between motherhood and recovery

The perinatal period was a time in which mothers were concerned about how their substance use might have impacted their child(ren), increasing their motivation to stop using substances. Upon discovering that they were pregnant, participants overwhelmingly expressed an urgent need and desire to abstain from substance use. Many stated that they were able to stop substance use immediately. For example, one participant stated

The moment I seen that I was pregnant on the stick, I had an eight-ball meth and I flushed it. I was willing. And like I didn’t touch anything, not anything. – Participant 031

Some participants discussed seeking treatment for their SUD when they found out they were pregnant to abstain from substance use and to protect their child. One participant explained that she was “worried that if I quit, I might have a miscarriage” whenever she found out she was pregnant, therefore she decided to enter treatment.^1^ However, some participants said that despite their strong desire to be abstinent when pregnant, they still struggled with substance use. One participant stated

It’s not something you can explain, why you know that you have a baby in you, but still you can put a needle in or why you know that your growing a child, but yet you can still do a drug. -Participant 010

Some described pregnancy and childbirth as times when their abstinence was at risk because of injuries that complicated the pregnancy or postpartum prescribing practices. For instance, in the following example, a participant described her experiences after giving birth.

Well, they tried to send me home with a prescription of Percocet. So I mean really did not shame me or nothing. I guess they just had a lack of knowledge because I refused that prescription because I had been clean for like 5 months. So, I thought that was really crappy and then they hand me a prescription like that knowing my history. – Participant 005

However, while pregnancy was a motivator to abstain from substances during pregnancy, participants reported that maintaining this abstinence was difficult postpartum and often resulted in a return to substance use. One participant explains the way being a mother and her disorder continuously conflicted with one another.

So it wasn’t that I cared for the drugs more, but my body had to have the drugs more where my mind and my heart wanted my kids more. So it was like a constant battle, a constant fight between my mind and my body on what to do. -Participant 029

Other participants explained that the severity of their disorder lead to a return to use, with one participant stating “drugs do weird things to your brain” when discussing her return to use during the perinatal period. However, another participant explained that her return to use postpartum was due to her believing she could no longer harm her child if it was not inside of her body, but would only be harming herself. She stated the following about her return to use.

“Just not having her inside my body, just thinking it’s just my body now. Before that’s what got me sober was that I was hurting, I could hurt her. So as soon as I didn’t think that was happening, I didn’t care for myself”.

### 3.2. Challenges being fully present as a parent

Many participants reported that when they were in active use, the disorder interfered with their ability to be present in their parenting role. Most participants described that they could provide basic needs for their children, such as food, clothing, and housing, but they could not fully meet their children’s emotional needs.

Whenever I didn’t have any drugs, you know I’m dope sick and my patience are short, you know or I didn’t have the energy to get out of bed. Or when I was eating Xanax like Tic Tacs, I couldn’t get up in the morning to get my kids. You know it impacted everything I did, everything. But I made it work, though, like I thought I did. Like I was there for my kids in physical form, but mentally and emotionally, I was not the parents that they deserved or needed. – Participant 007

Participants explained that this was not for a lack of interest or desire to be emotionally available but a consequence of the disorder. Specifically, they stated that they often were preoccupied with thoughts about how they would obtain substances to avoid experiencing symptoms of drug withdrawal. For one mother, drug preoccupation interfered with her ability to be an active parent to her children. While most mothers reported being able to be physically available for their children, this participant explained that she was not able to do so because of the symptoms of SUD.

I didn’t do what I should do as a parent. I didn’t cook. I didn’t clean. I just worried about getting the drugs. Spent money that I shouldn’t have spent. Didn’t spend time with them. My mind was just consumed with getting heroin. – Participant 010

Additionally, participants adamantly conveyed that they did not want their children to witness them using substances. Therefore, they would separate themselves from their children to engage in use as a means of protection. This often resulted in mother’s removing themselves from their children or using grandparents as alternate caregivers while they would engage in use. For example, one participant explained that she could not be the mother she wanted to be because she was protecting her children from witnessing her use.

I just feel like a mom should be you know not locked in a bathroom getting high while your kids are in the next room painting or you know being a mother is very hands-on. And that was never the mother I wanted to be. – Participant 012

Other mothers stated they would leave their children with the children’s grandparents when they would use. This was often for extended periods of time; in some instances, the children would move in with the alternate caregivers. One mother stated

I was gone most of the time because I didn’t want my kids to see me messed up…I didn’t want to have drugs around them. So, I would just leave them at my mom’s or his mom’s. Sometimes I wouldn’t come back for a week. – Participant 017

Another mother explained that she moved her children in with the children’s grandparents because her and her partner were actively using.

My first two had to get moved in with their grandparents and had to get use to all of that…they went a good while without seeing either one of us because we were out using. – Participant 009

### 3.3. Mothering within the government and legal system

Participants often cited negative experiences with child protective services (CPS), social services, and the legal system that complicated their ability to parent. Challenges with CPS were common among most participants, with the removal of their children often being a consequence of CPS investigations. Participants expressed that each case had different requirements that could be influenced by their previous personal relationships with CPS workers and court staff. Also, to regain custody of their children should they have lost custody prior, participants explained that there were many requirements they had to fulfill that often felt daunting and unobtainable, especially when they had limited support from family or social services. One participant expressed how having a SUD played a role in meeting these requirements by saying

You got to jump through this hoop, this hoop, this hoop, this hoop, this hoop. You know what I’m saying? To get her. And like when you’re a drug addict and you’re you know drug addicts want instant gratification. And so, when you give us this list of things like I mean, for a normal everyday person, those are easy… But for a drug addict, everything looks so far out of reach. – Participant 031

Additionally, participants expressed that they needed more guidance when navigating the different government and legal systems they were within. For example, one participant stated the following:

I really kind of relied on the court system to help me because I didn’t know what I needed. And I feel like the court system kind of failed me. They were like basically, well you figure it out you know? And I am just going to give you enough rope just to hang you, basically is how I felt. But I didn’t know, I didn’t know, and I genuinely didn’t know. – Participant 015

Some participants described a need for more support when it came to receiving social services that would help them provide for their children’s basic needs. One participant shared the following about her experience trying to obtain government benefits:

I was even told at one point I made three cents too much to get any support…and all I was wanting, I mean, it was just food stamps to make sure I had food for the kids. – Participant 002

### 3.4. Child protective services and separation

Participants frequently spoke of their children being removed from their custody or having fears of their children being removed by CPS. Some participants allowed other family members to care for their children before CPS legally separated them. However, others spoke of how CPS removed their children but did not always prioritize reunification or the children’s wellbeing. One participant explained that when CPS removed her child, it resulted in the child suffering worse consequences.

She used to go to school in dirty clothes for like four days in a row… She would have like shit in her underwear, just you know awful. Awful. They took her from me and gave her a horrible life. – Participant 031

The experience of having a child removed from their care was often described as traumatic for both the mother and the child. When the children were removed from their custody, especially if their parental rights were terminated, participants expressed that they felt they had no reason to abstain from substances. The removal of their children was a determinate to their recovery. One mother describes the following experiences after she said goodbye to her children:

I left straight from there and went two parking lots down where I was sure she wouldn’t drive past and see me and sat there and me and my boyfriend shot up heroin until like I couldn’t see straight. Like he had to drive us home. And it just got so much worse after that. Like I didn’t want to live anymore. – Participant 032

When mothers did regain custody, they often felt that they had to relearn to parent because they had been separated from their children and had not parented while abstaining from substances. For instance, one participant said the following:

There’s CPS involvement and your kids are gone. And then once they come back, your kind of, you’ve not parented in a year or two years or however many years. And then you know you’re with this child, and there’s a lot of disciplinary issues. – Participant 011

### 3.5. Impacts on children

Many mothers receiving substance use treatment believed that their substance use did have an impact on their children in numerous ways. One mother explains the various ways she felt her use impacted her children by saying:

They saw me at my worst. You know I never OD in front of them, but I have nodded out in front of them. I’ve been really high you know to the point where I forgot to feed them. You know I feel horrible saying that, but it’s true. You know sometimes drugs make you do things you don’t want to do. You know having strange men around just because of his free drugs, you know they saw that and they dealt with, you know, a lot of it. - Participant 028

Several participants explained that the cost of substances resulted in financial stress that negatively impacted the children. Some mothers expressed that their SUD influenced their mood, resulting in them having less patience and being more irritable when they could not access substances. At times, their use also changed their style of parenting. For example, one participant explained the following inconsistencies:

It made it to where I wasn’t consistent. I didn’t get along with my son when I wasn’t high, really. He thought I was mean, more mean, but not even necessarily just more mean. It was a consistent mean. You cannot jump on the couch. You cannot jump on the couch. It’s not okay. You know what I mean? But when I was high, I would not even notice them jumping on the couch for five minutes so they could do it. – Participant 024

Another way mothers believed their substance use impacted their children was through the environments in which their children grew up. Most participants explained that their children had witnessed household members under the influence of substances and overdoses, experienced death, saw intimate partner violence, and were victims of maltreatment from individuals who were intimate partners or others using substances in the household. One mother stated the following about her child finding her under the influence of substances:

I had blacked out at my mom’s house, and he come into the living room and got a needle out of my hand, and went and buried it in the yard and put me to bed. And the next day, two days later, when I got up, like he was pissed about it. And I get it now. – Participant 011

Another mother explained a conversation with her son following the death of her friends who had passed from overdose.

I’ve lost friends in addiction. You know what I mean? That overdosed on heroin or

something that would be close to him even. And he’d be like, “Well, how did they die, Mom?” You know what I mean? And I’m just like, “Oh, son. They do drugs.” You know what I mean? And he says, “Do I ever have to worry about you dying?” – Participant 024

Often the mothers were not aware of some of the circumstances their children faced, only learning of the experiences when their children told them later. The mothers reported feeling immense guilt about their children’s circumstances, took accountability for the circumstances, and longed to rebuild relationships on terms that were comfortable for their children.

Participants reported feeling that other’s negative views of them influenced how their children perceived them and their children’s emotional health. Some mothers expressed that their children hearing negative remarks resulted in their children having conflicting feelings about their mothers and damaged the mother-child relationships. At times participants also stated that this rhetoric left their children feeling alone. For example, one participant explained what her daughter experienced after she witnessed the participant’s nonfatal overdose:

“Nobody ever helped her through that. They just told her I was a junkie whore, and I was a piece of shit. So she wasn’t ever helped to get through that. So she’s hurting inside.” – Participant 027

### 3.6. Age-dependent consequences

Mothers reported that the age of their children during their active use was an important factor that influenced how the children were impacted. Many mothers with adolescent children reported that they felt their SUD put a strain on their relationship. The participants said that their adolescent children had expressed no longer wanting to speak to them because of what they experienced during their mother’s active use. They reported that their children were afraid to trust them again or did not want to witness their mother’s potential return to use. For instance, one mother said:

I would get out of jail or prison and tell her I was going to do good and not do drugs. And then I would go back to jail or prison and do drugs. And she couldn’t trust me or believe me. So she didn’t want anything to do with me. -Participant 010

Some mothers reported concerns about how using substances while their children were young may have impacted their attachment with their children. However, most mothers who had younger children reported feeling that their substance use did not influence their children because the children was too young to understand the circumstances. One participant explained that she entered treatment when her daughter was young to prevent her from witnessing her use. She stated:

That was my plan that I didn’t want her to remember me like or see me like that ever…I know how it affected me and I used to think about the places I had been in, I never wanted her to experience. Like some of the houses I’ve been in or you know some of the situations I’ve been in, I just never wanted to put her through that. I just know how awful and a mess I was and I just wouldn’t want her to see that. – Participant 006

Despite the challenges and barriers mothers and their children faced, going to substance use treatment was often seen as a way to mitigate any further harm and provide a better life for themselves and their children, as one mother said, “I went to rehab to be a better mom”.

## 4. Discussion

The findings of this study portray how substance use and parenting relate to one another in the lives of women receiving SUD treatment. This evidence adds to the literature by describing the ways in which maternal SUD may potentially impact parenting practices, motherhood, and children’s lives. Our findings expand on the relationship that has been found in epidemiologic studies between negative parenting practices and SUD. The results suggest that symptoms of SUD, such as drug preoccupation, act as a barrier to positive parenting. The participants SUD led them to being involved in many social services systems such as CPS which often resulted in separation that disrupted and ceased the parent-child relationship. The mothers in this study described four themes related to their SUD, motherhood and parenting, which were 1) battle between recovery and motherhood, 2) challenges being fully present as a parent, 3) parenting within government and legal systems, and 4) impacts on children. Taken together, the four themes illustrate the entangled and, at times, contradictory forces shaping the experience of motherhood among women with SUD. The *battle between recovery and motherhood* and the *challenges being fully present as a parent* are deeply connected, both reflecting the internal struggles mothers face as they attempt to prioritize their children’s needs while managing the physical and psychological demands of SUD. These internal conflicts are often compounded by external barriers captured in the theme of *mothering within government and legal systems*, where punitive and inconsistent institutional responses frequently undermine women’s efforts to achieve abstience and reunify with their children. In contrast, the theme of *impacts on children*—especially when viewed through the lens of *age-dependent consequences*—reveals a sobering disconnection: while mothers are highly attuned to their children’s suffering and often driven by a desire to protect them, the cyclical nature of substance use, trauma, and systems involvement frequently impedes this intention. Despite these tensions, a throughline of maternal concern and accountability is evident across themes, suggesting that interventions must not only address substance use but also recognize the structural, emotional, and relational dimensions of recovery and parenting. Supporting mothers in healing these multiple layers of disconnection may be essential for fostering long-term recovery and improving family outcomes.

For women with SUD, becoming a mother often represents a pivotal point that bears increased motivation to abstain from substance use, as was the case for all the study participants. Yet, many of the 35 mothers that were interviewed for this study reported returning to substance use after childbirth. This return to use could contribute to engagement in poor parenting practices found among mothers with SUD. Return to use is common among mothers with a history of substance use. One study found that 80 % of mothers who achieved abstinence during pregnancy returned to use postpartum (Forray et al., 2015). Previous studies have found various factors that put mothers with SUD specifically at higher risk for return to use, such as, a history of childhood adversity, particularly childhood sexual abuse, comorbid mental health disorders, medical complications during pregnancy, postpartum pain, and poverty (Halpern et al., 2018; Nawaz et al., 2022; Rankin et al., 2023). Many of these risk factors were also prevalent among the women in our sample, with the average ACE score being 5.4 showing substantial experiences of childhood adversity, more than half of the participants reporting childhood sexual abuse, and a current household income lower than $25,000 annually. The commonality of postpartum return to use also poses a risk to their children as return to use is associated with child maltreatment (West et al., 2020). Despite evidence highlighting the need for services that specialize in recovery for pregnant and parenting women, service availability remains low across the US, especially in rural areas such as the majority of WV from which this study sample is drawn (Kramlich et al., 2018; Meinhofer et al., 2020). To promote positive parenting more support is needed during the perinatal period to prevent a return to use that can negatively impact parenting.

The participants in this study had frequent encounters with government and legal systems, especially the child welfare system. Mothers cited negative experiences with these systems that they reported had adverse effect on their parenting and may even have served to increase their risks for continued substance use. Primarily, mothers reported that the social services systems they were involved in contributed to them being separated them from their children, prohibiting them from being active parents. Once they had been separated from their children, most participants no longer felt a desire to remain abstinent and returned to use, at least partially to cope with the separation.

Many mothers reported that they did not meet the requirements to receive governmental assistance, especially if they were working. However, they also stated that their jobs did not provide enough income to support their children. Providing basic needs for their children was reported to be a priority for the mothers in our sample. However, obtaining government assistance that would allow them to do so was often reported to be challenging. Limiting access to government assistance could negatively impact this population as low socioeconomic status has been linked to return to use and reduced chances of family reunification (Lloyd, 2018; Panebianco et al., 2016). Involvement in the court systems led to confusion for many participants in the study, as they relied on the court’s guidance but often felt that they did not receive any. Some suggested that without this guidance they could not meet the requirement and stipulation put on them to regain custody of their children, prohibiting them from taking on the role of primary caregivers. Participants further explained that these systems often made them feel defeated and hopeless which they felt contributed to a return to substance use.

All study participants discussed the child welfare system extensively. Most mothers in the study also explained that the removal of their children stopped them from being children’s primary caregiver meaning that they felt unable to adequately parent their children. Parental substance use is often a concern in the majority of neglect allegations within the child welfare system (Palmer et al., 2024). It has been estimated that parental SUD impacts 50–80 % of families involved in the child welfare system (Marsh et al., 2011). Negative experiences from the child welfare system described by the mothers in this study have been found in multiple other quantitative and qualitative studies. Other research has found that many practices by the child welfare system may have negative consequences for mothers with SUD and their children, such as mothers avoiding services due to fear of CPS and child removal, higher incidence of mental illness in mothers after removal, and attachment disorders in children (Barnett et al., 2021; Colvin and Howard, 2022; Goldsmith et al., 2004; Trivedi, 2019; van Bijleveld et al., 2015; Wall-Wieler et al., 2017). One alarming finding from our study was that the removal of children can decrease motivation for abstinence and increase risk of substance use. This finding aligns with the results by Thumath et al., that found that mothers who had a child removed from their home experienced a 55 % increase in the odds of nonfatal overdoses (Thumath et al., 2021). Mothers in substance use treatment who are involved with the child welfare system often have needs for multiple services, such as those that address survival needs for the mothers and children, childcare challenges, mothers’ past trauma experiences, and children’s attachment challenges, - all that ideally should be incorporated into substance use treatment to improve outcomes for this population (Channell Doig et al., 2023; Grella et al., 2006; Marsh and Smith, 2011). To demonstrate, a recent review and meta-analysis found that mothers who used subtances and who participated in integrated programs were less likely to have their children removed and reduced their levels of substance use (Neo et al., 2021).

Mothers in our study described challenges in parenting where they were not able to be emotionally available for their children because of their substance use. A major symptom of SUD is drug preoccupation, which can divert mothers’ attention from their children and interfere with their ability to be emotionally available (Milligan et al., 2020). Poverty is a common coexisting factor in the lives of mother with SUD. This can contribute to limited emotional availability and was reported by several mothers in this study. Research shows that poverty exasperates parenting challenges (Beasley et al., 2022). For example, a recent study found that mothers with SUD who had multiple socioeconomic risk factors such as unemployment and housing issues were less likely to be reunified with their children after CPS separation (Lloyd, 2018). The neurobiology of how substance use impacts the regions of the brain responsible for parenting can also help explain this phenomenon. The parts of the brain responsible for parenting are overtaken when substances are used in a way that makes maintaining the use a priority (Porreca et al., 2018). This leads to decreased reward sensitivity and higher levels of stress related to parenting (Bosk et al., 2019; Porreca et al., 2018). In other words, for many of the mothers, parenting feels less rewarding than the relief from substance use because of how the region in the brain is coopted by the chemical reward circuits. While this can impact the children of mothers with SUD, other environmental and household-related factors can also negatively impact their well-being. Most study participants explained that their substance use exposed their children to environments and relationships that were potentially traumatizing. Many of these risks, such as parental separation and witnessing intimate partner violence have been categorized as ACEs, which are associated with poor academic achievement, depression, anxiety, and negative physical health conditions in children (Qu et al., 2024; Turney, 2020). Other consequences of more drug-specific experiences described by participants in our study, such as witnessing an overdose and exposure to drug paraphernalia, remain understudied.

The findings from this study have various implications for intervention development. First, interventions and services should be developed that focus on keeping the family unit together while supporting mothers with basic needs. Our findings did not suggest a lack of parenting competence by most participants, rather lack of resources and opportunities to engage in their role as parents. Additionally, based on the mother’s perspective of their children experiences, trauma-specific and attachment-based services would be helpful for their children. In this study, many mothers explained that their children had been exposed to various forms of trauma, including parent-child separation, that could negatively impact the attachment relationship. Mothers also reported that such trauma exposure at times resulted in their children being frustrated and distrusting of their mothers. While evidence regarding the negative impact maternal substance use can have on children continues to grow, children are often not the intervention target, and few studies evaluating parenting programs measure children’s outcomes (Calhoun et al., 2015). The programs that do exist for children have shown some improvements in children’s outcomes. However, they often are not integrated into substance use treatment programs for their mothers, where they could be operated in tandem with the mother’s substance use treatment and parenting interventions, potentially increasing effectiveness for both parties (Bröning et al., 2012, 2019; Lewis et al., 2015). More research is needed to develop and test such interventions as they are limited, only engage the caregiver, or are solely tailored for parents who have young children (Arria et al., 2012; Bosk et al., 2019; Bröning et al., 2012; Guyon-Harris et al., 2023).

### 4.1. Limitations

The findings of this study should be interpreted while considering certain limitations. Our study was conducted with a predominately White sample. Those who identify as other races may have different perspectives and experiences. Also, participants were asked to report about their children’s experiences, which could likely introduce social desirability and/or recall biases. We also did not observe if participants were actively parenting their children at the time of data collection which may have impacted their reporting. Further, our sample was limited to mother who were in recovery from SUD at the time of data collection; those still in active use may have provided differing perspectives. The sample was also predominantly from mothers who had severe cases of Opioid Use Disorder and Methamphetamine Use Disorder, which may limit the applicability to mothers who have less severe forms of SUD. Lastly, this research was conducted without the use of community-engaged approaches.

## 5. Conclusions

This study described the inter-influence of parenting and substance use among mothers receiving SUD treatment. The role of motherhood and the symptoms of SUD are interrelated and should not be considered in isolation. While pregnancy was a time of increased motivation for abstinence from substance use, mothers in this study bring attention to the need for continued support throughout the perinatal period as return to use was common. Mothers explained that their SUD acted as a barrier to meeting their expectations of motherhood while also conveying that the governmental support systems they were involved in made parenting more challenging. In addition, the adversity experienced by children of mothers receiving SUD was highlighted in this study as reported by the mothers. Integrating services into treatment programs that address the specific needs of mothers, and their children simultaneously is necessary to serve this population better and minimize negative consequences. Additionally, the results of this study also have policy implications that suggest stringent CPS policies could negatively impact the mother child dyad.

## Figures and Tables

**Table 1:** Characteristics of study participants N = 35.

Variable			
	
Categorical variables	n		%

**Education**			
High school graduate or less	11		31.4 %
Any Higher Education	24		68.6 %
**Income**			
> 25,000	17		48.6 %
< 25,000	18		51.4 %
**Race**			
White	32		91.4 %
Other	3		8.6 %
**Primary Substance of Choice**			
Opioids	22		62.9 %
Methamphetamines	10		28.6 %
Cocaine	2		5.7 %
Alcohol	1		2.6 %
Continuous variables			SD
Age	35.94	7.10	
Number of Children	2.49		1.54
ACE Score	5.43		2.79
Mean ACE Score of Child(ren)	3.42		2.28

**Table 2:** Types of adverse childhood experiences endorsed by study participants (N = 35).

ACE Type	Study Participants (%)

Emotional Neglect	42.9 %
Emotional Abuse	57.1 %
Physical Neglect	20.0 %
Physical Abuse	51.4 %
Sexual Abuse	57.1 %
Divorced or Separated Parents	85.7 %
Household Mental Illness	57.1 %
Household Substance Use	77.1 %
Household Incarceration	42.9 %
Domestic Violence against Mother/Stepmother	51.4 %

**Table 3:** Qualitative findings on how SUD impacts parenting and children.

Theme	Supportive Quotes

**The Battle Between Motherhood and Recovery**	“When I first became pregnant, I was really excited and I immediately quit doing pills... really wanted to be a mom. And I stayed sober for four years after I had him.” -Participant 033 “I was scared. Very happy because I’ve always wanted to be a mom. But most of it was scared because I didn’t want to relapse. Participant 006 “Here I am sticking a needle in my arm with a baby in my belly. And your mind is just I was emotionally detached. Like I loved my kids, but I couldn’t stop using. I was a shitty mom. You know it was shitty to put my needs above my kids. And I knew deep down that was not who I was. But drugs do weird things to your brain.” -Participant 012 “I had no clue what I was going to do because you know it’s just not logical to think that you can just lay it down and not pick it back up. When you’ve been in active addiction on and off for you know 14 years, you know that’s not true. You know and I hate to say this because I never see myself as somebody that would use while pregnant. But I found out you know I ate my words because even though you’re pregnant and you know you’re pregnant, you know that sickness is - your addiction doesn’t care.” –Participant 025
**Challenges Being Fully Present as a Parent**	“I would leave my kids with my grandparents so I could go find drugs to have, to not be sick. And then when I had the drugs, I still didn’t help that much because I was looking for them every day to not be sick.” -Participant 026 “I mean, there’s stuff I could have done that I didn’t do. I could have spent more time with them than I did. I could have been there for them. You know, you know what I mean? Being a better parent than instead of always hiding in the bathroom or you know not taking care of them right.” -Participant 028 “I was in the bathroom a lot and they was always wanting in the bathroom and I wouldn’t let them in. So like looking back like I feel bad about that.”-Participant 005
**Mothering within the Government and Legal System**	“You know, they put a lot of stipulations. Like In order for me to be able to get overnight visits with my daughter and stuff, like I have this list of stipulations that I have to acquire before I can even go up to petition for her overnight visits. You know, They want you to do all this stuff. And I don’t disagree with any of the things they want me to do at all. But you know some of that stuff you can’t do.” – Participant 020 “At the end of my CPS case, my parenting worker even told me she said, “He should have never been taken from you if you was at (domestic violence shelter) and stuff like that” – Participant 013 “there’s things that you could have put in place some type of like instead of just immediately taking their children and giving them no reason to live or no reason to get clean. No reason you know because you’ve already took them. What’s the point of getting clean then? You know? That’s how addict thinks it’s an addict mind” – Participant 029
**Impacts on Children**	“I do not have a relationship with my middle one. He can’t forgive me for the relapses, for the things that I’ve done, things that I’ve said, places I’ve went, people I went back to, relationship-wise, this and that and the other” – Participant 029 “But with my middle son, I only had him for four months before we gave him to them. So I don’t feel as close to him” – Participant 009 “He’s seen the worst out of all my children. He has seen the most and seen the worst side of me and what drugs can do. He sees me go through many, multiple terrible relationships, physical abuse, emotional abuse. You know and he’s gotten some of that as well from my partners. And today, we’re okay. Like I’m involved in his life and in my grandchildren’s life. There were many years that I was cut off, and I had no contact with him. I had no contact with his kids. And I get that.” – Participant 011 my daughter is extremely intelligent. She’s smart, and she’s got a good head on her shoulders, and I respect her for that... she said, “you know I don’t want anything to do with you until you have a year clean.” And I said, “I can respect that. You know That’s fine.” – Participant 007

## Footnotes

1 The American College of Obstetricians and Gynecologist (ACOG) and the Substance Abuse and Mental Health Services Administration (SAMHSA) recommend treating pregnant women with Opioid Use Disorder with methadone or buprenorphine and behavioral therapy.

## Appendix A. Interview Guide

### Interview Guide

1. Tell me about yourself, specifically describe your childhood.
  - Probe: household environment, adverse childhood experiences, support systems
2. Describe how you were first introduced to substances?
  - Probe: Age? Trajectory? Introduced by Whom?
3. How, if at all, did substance use influence you becoming a mother?
  - Probe: Age? Trauma related to becoming a mother?
4. When you were pregnant for the first time, how did you feel?
  - Probe: Experiences with pregnancy (always probe for substance use)
5. When you gave birth for the first time, what did you experience emotionally?
  - Probe: (Fear? Excitement? Hope?)
6. As your children aged, what were your experiences as a mother like?
  - Probe: Relationship and attachment to child? Feeling about being a mother? Parenting Stress?
7. Do you feel your substance use had any effect on your child(ren)? Explain
  - Probe: Do you feel your child needs any type of treatment or services? What would you like to see?
8. Did you receive any support at your treatment program that helped you cope with past challenges or trauma? For instance, a parenting support group or family counseling? If so could you tell me more about that?
  - Probe: How did this help? Or what would be helpful? Interventions for family and children? Help coping with triggers or facing trauma?
  - Probe: What supports do you believe there should be for mothers in recovery and their children.
